# 

**Published:** 1913-06-15

**Authors:** 


### ANNOUNCEMENTS.

July Meetings: New Jersey State Dental Association, Asbury Park, July 16th to 18th.

Montana State Board of Examiners, meets July 14th to 17th, at Helena

Idaho State Board of Examiners, meets at Boise, July 7th.

The National Association of Dental Examiners, will meet at Kansas City, Mo., July 7th and 8th.

The National Association of Dental Faculties, will meet at Kansas City, July 4th.

Southern Branch of the National Dental Association, will meet at Old Point Comfort, July 22nd to 25th.

National Dental Association, will meet at Kansas City,

Mo. July 8th to 11th. Dr. C. C. Allen, 507 Rialto Building, Kansas City, is chairman of the Local Committee of Arrangements.

Wisconsin State Association, meets at Madison, July 8th to 10th, 1913.

West Virginia State Dental Society. The Seventh Annual Meeting of The West Virginia State Dental Society will be held in the Assembly Room of The Chancellor Hotel, Parkersburg, W. Va., August 13-14-15, 1913. Opening session at 2 o'clock, Wednesday, August 13th.

Our *new Program Committee* have all thrown their *Hats in the ring*, and they promise the *Best Program* ever presented to our Society.

The Executive Council have directed me to urge *every member* to be present, and if possible bring a *new* member with you. Mark off, (from your appointment book), these days of meeting. *Do it now.* You will learn *something* there, and *we need you.* The place of meeting, The Chancellor Hotel, Parkersburg; The dates, August, 13-14-15.

Come, you will find the time well spent, the money (for the trip), well invested.

Frank L. Wright, *Secy.*

Fourth International Congress on School Hygiene. To the Editor of Dental Register, Dear Sir:—

May we not depend upon your editorial aid at this time in contributing to the success of The Fourth International Congress on School Hygiene, which is to be held in Buffalo August 25-30th inclusive, under the patronage of the Honorable Woodrow Wilson.

We desire to bring together a record number of men and women interested in improving the health and efficiency of school children, moreover to make this Congress—the first of its kind ever held in America—one of direct benefit to each individual community. Such a thing is made possible only by the hearty co-operation of editors in their various publications.

There is now being arranged a comprehensive program of papers and discussions covering the entire field of school hygiene. There will be scientific exhibits, representing the best that is being done in school hygiene, as well as commercial exhibits of practical and educational value to school people. Nor will the entertainment of the delegates in any way be a minor feature. Plans are being made for a series of social events, including receptions and a grand ball, a pagent in the park, and excursion trips to the great industrial plants of Buffalo, as well as to the wonders of Niagara Falls, and the Rapids. Buffalo itself has just taken up a collection of $40,000, for the purpose of covering the expense of the Congress.

Delegates will attend from all the leading nations, from every college and university of note in this country, and from various other educational, scientific, medical and hygienic institutions and organizations. The Congress is further open to all persons interested in school hygiene. Membership may be secured on the payment of a five dollar fee. Applications should be sent to Dr. Thomas A. Storey, College of the City of New York, New York City.

It is greatly desired to secure a large membership of the congress, and to this end, may we not count upon you in spreading the news of the congress and in calling attention to the benefits following the presence of all those actively engaged in promoting the welfare of the child, the school, and the community?

The man of to-morrow depends upon the child of to-day, and the child of to-day, roughly speaking, spends half of his waking hours under the influence of school conditions. Are you interested in making these conditions what they ought to be? If you are, give this Congress publicity. That is one way in which you can help.

Cordially yours,

Thomas A. Storey, *Secy.*
College of the City of New York,
New York City.

Oral Hygiene at Buffalo. "Oral Hygiene" will occupy a prominent place on the program of the Fourth International Congress on School Hygiene, which will be held in Buffalo, the last week in August. A special symposium is now being arranged under the direction of the National Mouth Hygiene Association. Papers will be read upon the subjects of "Dental Inspection." Dental Dispensaries," "Dental Clinics in the public schools," and various other topics of special interest to the dental profession.

One of the exhibits will be the motion picture films, entitled "Tooth Ache," which is produced under the auspices of the National Mouth Hygiene Association.

Dr. D. E. Jessen of Strassburg, Germany, the father of the movement for care of school children's teeth is expected to be present and give an address on this subject. His work has made him an authority on this subject and he will undoubtedly have an interesting address.

---

National Mouth Hygiene Association's Second Annual Meeting. The Second Annual Meeting of the National Mouth Hygiene Association will be held in Kansas City, Mo., July 9—12, 1913.

The Board of Governors will meet at the Hotel Baltimore at 9 A. M. on July 9th, 10th, and 11th, to consider questions to be presented to the general body. These meetings are open to members of the Association. Those having matters which they wish to present to the Association for consideration must present them to the Board at one of these meetings.

The regular meeting of the Association consists of three sessions: Friday at 1.30 P. M.; Friday at 8.00 P. M.; Saturday at 9.00 A. M. (open to the general public.)

A very interesting and instructive literary program is to be presented.

The Association has charge of the Mouth Hygiene Section of the Fourth International Congress on School Hygiene, and the Board of Governors' plan is to have, in so far as possible, the program duplicated at both the National Meeting and the Congress.

The most important subject to be presented to the Association will be the question of "Ways and Means for Conducting the Campaign of the Association in its General Presentation of Mouth Hygiene to the Public". A complete, pratical economic and co-operative plan which will permit every section of the country to begin an active appeal to the general public will be presented for consideration. It is desirable that every member be present when this important subject is presented for discussion.

W. G. EBERSOLE,
Secretary-Treasurer,
800 Schofield Bldg.,
Cleveland, Ohio.

---

NATIONAL DENTAL ASSOCIATION. All reputable practitioners of dentistry and medicine are cordially invited to attend the 1913 session of the National Dental Association, which will be held in Kansas City, Mo., July 8th to 11th.

This will probably be the most important meeting in the history of this Association, owing to the fact that all the State Dental Societies, that have met since the Washington meeting, have voted to become Constituents of the National.

The Officers and Committees have prepared an interesting program, but it is impossible to incorporate the Clinical Program in the Journal announcements; however the Clinical Committee does not expect to present the number of Clinics which have been listed for the past few years, but have planned to present a smaller number, which are classified so that they will be most interesting.

The following is the Literary Program: President's Address, Frank O. Hetrick, Ottawa, Kan.; Scientific Foundation Fund, Weston A. Price, Cleveland, O.; Orthodontia and its Relation to Dentistry, Roscoe A. Day, San Francisco, Cal.; Crown and Bridge Work, J. L. Howell, Denver, Col.; The

Missing Steps in Platework, Gail W. Hamilton, Council Bluffs, Ia.; The Saliva, Percy H. Howe, Boston, Mass.; Conflicting Opinions Concerning the Manufacture and Use of Alloys for Dental Amalgams, Marcus L. Ward, Ann Arbor, Mich.; A Preliminary Report on the Action of AS2 O3, Herman Prinz, Philadelphia, Pa.; Physiological Action of Nitrous Oxide-Oxygen, Analgesia and Anesthesia, Carl G. Parsons, (M. D), Denver, Col.; Something of the Etiology and Early Pathology of the Diseases of the Peridental Membrane, With Suggestions as to Treatment, Arthur D. Black, Chicago, Ills.; The Value of the Radiograph in the Practice of Modern Dentistry, Howard R. Rapier, Indianapolis, Ind.; Dental Educational Harmony, G. S. Junkerman, Cincinnati, Ohio; Prophylaxis, Illustrated with Lantern Slides, Burton Lee Thorpe, St. Louis, Mo.; The Etiology and Progress of Dental Caries, Edgar D. Coolidge, Chicago, Ills.; The Application in Practice of what is Known Concerning the Diagnosis and Treatment of Diseases of the Dental Pulp, Harry B. Tileston, Lou'sville, Ky.; Periapical Infection, Clarence J. Grieves, Baltimore, Md.

*Railroad and Hotel Information:* The Central Passenger Association, the Western Passenger Association and the Southwestern Passenger Association, have granted an open rate of 2 cents per mile in each direction in their territory, with a minimum excursion fare of $1.00. Tickets on sale the 5th to 8th of July and good returning up to July 20th. The Trunk Line Association has declined to make any concessions. Additional information can be secured from any local railroad agent.

A list of hotels, and their rates, have been published and we save space in this announcement by referring you to the June Journals. Those anticipating attending this meeting should promptly make reservation, if this has not already been done.

FRANK O. HETRICK, *Pres.*,
Ottawa, Kan.
HOMER C. BROWN, *Rec. Sec.*,
185 East State St., Columbus, Ohio.

